# A Cross-Sectional Study of Factors Influencing Pre-Operative Anxiety in Orthognathic Patients

**DOI:** 10.3390/jcm12165305

**Published:** 2023-08-15

**Authors:** Xiu Ling Florence Kok, Jamie Gwilliam, Mark Sayers, Elinor M. Jones, Susan J. Cunningham

**Affiliations:** 1 Department of Orthodontics, UCL Eastman Dental Institute, 21 University Street, London WC1E 6DE, UK; s.cunningham@ucl.ac.uk; 2 Department of Orthodontics, St George’s Hospital, St George’s University Hospitals NHS Foundation Trust, London SW17 0QT, UK; jamie.gwilliam@nhs.net; 3 Department of Orthodontics, Queen Mary’s Hospital Sidcup, King’s College Hospital NHS Foundation Trust, Sidcup DA14 6LT, UK; mark.sayers@nhs.net; 4 Department of Statistical Science, University College London, London WC1E 6BT, UK; elinor.jones@ucl.ac.uk

**Keywords:** orthognathic surgery, jaw surgery, anxiety, social support, resilience, coping

## Abstract

Orthognathic treatment is an important treatment modality to manage severe dentofacial discrepancies. Patients awaiting orthognathic surgery often experience increased anxiety, which may adversely affect post-operative recovery and treatment satisfaction. This study investigated the effects of a number of factors on pre-operative anxiety in orthognathic patients. Seventy patients prospectively recruited from three orthognathic centres in the UK completed a pre-operative questionnaire that included validated scales for measuring anxiety, social support, resilience, and coping styles. Sociodemographic data and satisfaction with the information provided by the clinical team were also elicited from the questionnaire. Univariable analysis showed that social support from a significant other (*p* = 0.026), resilience (*p* < 0.001), and satisfaction with the information provided by the clinical team (*p* = 0.002) were significantly associated with reduced anxiety, whilst avoidance coping (*p* < 0.001) and coping through seeking social support (*p* = 0.006) were significantly related to increased anxiety. With the exception of coping by seeking social support, these relationships retained significance in a multivariable regression analysis. Neither gender nor ethnicity moderated the effects of social support on pre-operative anxiety. These findings suggest potential avenues for clinicians to address with future interventions to reduce pre-operative anxiety. Further qualitative research may provide greater clarity on the relationship between these variables and anxiety.

## 1. Introduction

Orthognathic treatment is a treatment modality used to manage severe dentofacial discrepancies; it involves a combination of orthodontic fixed braces to correct the position of the teeth and maxillofacial surgery to correct the position of the jaws. The whole treatment process usually takes around 2–2.5 years, with the surgery conventionally taking place later in the process. This is important as it provides time for strategies to be implemented to manage pre-surgical anxiety. The existing orthognathic literature often focuses on outcome measures such as post-operative recovery and quality of life. However, the impending surgery may have considerable psychological impact, and research suggests that patients awaiting orthognathic surgery experience increased anxiety [1,2,3] so this is also an important aspect to consider.

Increased pre-operative anxiety has been associated with slower recovery and more post-operative symptoms in other forms of elective surgery [4,5,6]. Identifying factors that may reduce patient anxiety before surgery would therefore be potentially beneficial. Three factors that have been explored in relation to anxiety in the wider surgical literature were selected as the focus in this current study: social support, resilience, and coping styles.

Social support refers to the provision of physical, psychological, and/or financial help by a network of family, friends, or the community in times of need. Socially supportive relationships can positively impact on both physical and psychological health [7,8,9], and the results from a recent systematic review suggested an association between stronger social support and reduced pre-operative anxiety in elective surgery [10]. It is, at present, unclear if a similar relationship exists for orthognathic patients, where the treatment duration, process, and experience may differ from other elective surgical procedures. 

Resilience is defined as an individual’s ability to cope successfully with significant change, adversity, or risk. The available evidence suggests that high levels of resilience may be protective against anxiety, allowing resilient patients to have lower anxiety and demonstrate more effective coping strategies [11]. In one study, patients with high resilience exhibited significantly lower anxiety prior to arthroscopic knee surgery than those with low resilience [12]. To date, there is limited information regarding the role of resilience in orthognathic patients. 

There are many ways in which individuals may cope with stressful situations, and results in the literature are varied with respect to which forms of coping work better in a surgical setting. Some studies have suggested that problem-focused coping is associated with better recovery and mental health [13], whilst avoidance coping has been linked to reduced pre-operative anxiety in some types of surgery [14,15]. How orthognathic patients cope with the stress of their forthcoming surgery and whether this has any effect on anxiety is not yet clear. It would be helpful to determine if there is potential value in providing specific coping advice to patients in the lead up to surgery. 

This study, therefore, aimed to investigate the effects of social support, resilience, and coping styles on pre-operative anxiety in orthognathic patients, accounting for other factors, such as demographic variables and satisfaction with the information provided by the clinical team.

## 2. Materials and Methods

Ethical approval was obtained from the London-Bromley Research Ethics Committee and the Health Research Authority (REC: 19/LO/0992). Participants were recruited from the Royal National ENT and Eastman Dental Hospitals, UCLH NHS Foundation Trust (EDH), St George’s Hospital, St George’s University Hospitals NHS Foundation Trust (SGH), and Queen Mary’s Hospital Sidcup, King’s College Hospital NHS Foundation Trust (QMH). Patients who were eligible to participate were recruited from August 2019 to August 2022. There was no previous data in this area on which to base a sample size calculation as, to the best of our knowledge, this is the first study to explore this combination of factors in orthognathic patients. The sample size was based on the number of patients treated in the 3 units in an appropriate timescale. The criteria for inclusion were adult orthognathic patients (aged ≥18 years) who could understand and communicate in English, and were ready for surgery. Patients with craniofacial syndromes, those who had undergone previous orthognathic treatment, or ‘surgery first’ patients who had surgery in the initial stages of the treatment process were excluded as their experiences might have been different. All patients were informed that participation in the study was voluntary and a decision not to take part would not affect their treatment in any way. 

Patients completed the online questionnaire within 8 weeks prior to their scheduled surgery date and each questionnaire was coded to protect confidentiality. The questionnaire included four validated and psychometrically tested self-report instruments that assessed anxiety, perceived social support, resilience, and coping styles. There were also additional sections eliciting sociodemographic information, type of planned surgery, and satisfaction with the information provided by the clinical team. 

*State and Trait Anxiety*: The State-Trait Anxiety Inventory [16] is generally recognised as a ‘gold standard’ tool for evaluating anxiety, and was used to measure both state (STAI-S) and trait (STAI-T) anxiety. State anxiety is a transient emotional state of anxiety and tends to fluctuate over time as a function of the level of perceived threat, whereas trait anxiety refers to a person’s general tendency to feel anxious in response to perceived environmental threats and is purported to be relatively stable. State anxiety was most relevant for this study; hence, STAI-S was the outcome variable. The STAI scales are each composed of 20 items rated on a 4-point scale, with higher total scores denoting greater anxiety. In this study, a 3-level categorisation was also utilised, with scores ≥ 50 representing high anxiety, and those ≤30 reflecting low anxiety [2]. The decision to utilise a general anxiety scale, in preference to a surgery-specific anxiety scale, was made on the basis that this study intended to explore anxiety in the approach to surgery, where there may be other potential stressors that can contribute to anxiety, in addition to the surgical procedure itself. Surgery-specific anxiety questionnaires tend to focus mainly on anxiety related to the actual procedure and/or anaesthesia.

*Social support*: The Multidimensional Scale of Perceived Social Support (MSPSS) is a 12-item instrument scored on a 7-point scale that assesses the perceived adequacy of support from 3 sources: Family, Friends, and Significant Other [17]. The scores are summed and then divided by the number of items in the scale/subscale, with a score of ≥5.1 suggesting high social support, whilst scores between 3 and 5 indicate moderate support [18]. 

*Resilience*: The Connor–Davidson Resilience Scale 10 (CD-RISC 10) is a measure of resilience that consists of 10 items scored on a 5-point scale, with 0 reflecting ‘*not true at all*’ and 4 as ‘*true nearly all the time*’. Total scores for the CD-RISC 10 range from 0 to 40, with higher scores denoting greater resilience [19]. 

*Coping styles*: The Brief COPE consists of 28 items representing 14 subscales of coping and rated on a 4-point scale with responses ranging from ‘*I haven’t been doing this at all*’ to ‘*I’ve been doing this a lot*’ [20]. The 4-factor higher-order classification of the subscales by Baumstarck et al. [21] was used to aid analysis, the factors being: coping through seeking social support, problem solving, avoidance, and positive thinking. 

Statistical analysis was performed using SPSS for Windows (IBM SPSS Statistics for Windows, Version 28.0. Armonk, NY, USA: IBM Corp.). Descriptive and inferential analyses were used to provide an overview of participant characteristics and evaluate the individual and combined effects of the independent variables on pre-operative anxiety. Tests included the Mann–Whitney U test and univariable and multivariable regression analyses. Based on the findings in a systematic review by Kok et al. [10], the moderation effects of gender and ethnicity on the relationship between social support and anxiety were also determined through the computation of an interaction variable that was inputted into a regression. Statistical significance for all tests was set at *p* < 0.05.

## 3. Results

### 3.1. Participant Characteristics

Seventy-five patients consented to take part in the study. Three patients did not subsequently complete the questionnaire due to COVID-related disruptions and 2 patients decided not to proceed with surgery, thus the final sample consisted of 70 patients. 

Of the 70 patients who completed the questionnaire, 57.1% were female, the mean age was 23.75 years (SD: 6.56, range: 18–55 years), and 47.1% were White. Most of the participants (92.9%) were single and were either students or working adults, with 51.4% holding degree level qualifications. More patients were scheduled for bimaxillary surgery (64.3%) than single jaw surgery (Table 1). The majority of patients (75.7%) presented with a Class III malocclusion (where the lower teeth/jaw protrude relative to the upper teeth/jaw), 20% of the patients had a Class II malocclusion (where the upper teeth/jaw appear more protrusive), and only 4.3% were Class I (where the antero-posterior relationship of the jaws is acceptable but patients had vertical discrepancies and/or facial asymmetry). Patients scored how satisfied they were with the information provided to date in their treatment and were generally highly satisfied with the information relayed by the clinical team (median score of 15 out of 15). Table 1 shows the STAI-S scores according to participant characteristics.

The median STAI-S score for all 70 patients was 35 (mean: 36.70 ± 11.27), and 64.3% of the sample were classified as being either moderately or highly anxious. Median state anxiety for females was higher than for males, with a relatively small *p*-value related to this difference (*p* = 0.068). There were no significant differences in state anxiety distributions based on ethnicity, education level, employment status, type of malocclusion, or type of jaw surgery. It should be noted that in view of the small number of Class I participants (*n* = 3), the analysis of anxiety in relation to malocclusion was only conducted for Class II and III patients. Similarly, for employment status, the analysis only compared anxiety in students with those in employment due to the small number of participants (*n* = 7) who were not currently employed. The relationship between state and trait anxiety was also evaluated, using a linear regression model. Heightened state anxiety was significantly associated with greater trait anxiety (B = 0.645, *p* < 0.001) and the median STAI-T score was 38, which reflected moderate trait anxiety.

The MSPSS scores showed that participants felt well-supported, with 75.7% considering themselves to have high levels of social support and only 1.4% reporting low support. Similar scores were noted across the three subscales (Table 2). The median CD-RISC 10 score was 29. Raw scores from the Brief COPE are presented in Table 2; they were also subsequently standardised across the four categories to allow comparison of the utilisation of different coping styles. Based on the standardised scores, positive thinking was used most commonly, followed by problem solving and seeking social support, with avoidance coping being the least commonly used.

### 3.2. Relationship of Independent Variables with State Anxiety

The presence of social support from a significant other (B = −2.194, *p* = 0.026), higher levels of resilience (B = −0.818, *p* < 0.001) and satisfaction with the information provided by the clinical team (B = −2.556, *p* = 0.002) were all significantly associated with reduced anxiety. In contrast, increased utilisation of coping through seeking social support (B = 0.721, *p* = 0.006) and through avoidance (B = 1.587, *p* < 0.001) were significantly associated with increased state anxiety (Table 2). 

Inclusion of an interaction variable into a regression analysis exploring the relationship between social support and pre-operative anxiety revealed that neither gender (*p* = 0.147) nor ethnicity (*p* = 0.077) appeared to impact significantly on this relationship, with *p* values greater than the pre-defined criteria of *p* < 0.05.

**Table 2:** Univariable analysis of the relationship between the independent variables and pre-operative state anxiety.

Outcome Variable: STAI-S	Median	Mean (SD)	Scale Range	B	*p*-Value
**Gender**				4.375	0.109
*0 = Male, 1 = Female*					
**Age**				0.345	0.095
**Ethnicity**				−5.039	0.061
*0 = White*					
*1 = Other ethnic groups combined*					
**Education level**				−1.098	0.687
*0 = Non-university education*					
*1 = Degree level education*					
**Employment status**					0.241
*Reference category: In employment*					
Student				−4.810	0.113
Not currently employed				0.952	0.836
**Type of malocclusion**					0.738
*Reference category: Class III*					
Class I				5.270	0.438
Class II				0.389	0.910
**Type of jaw surgery**				−1.151	0.685
*0 = Single jaw surgery*					
*1 = Bimaxillary surgery*					
**Satisfaction with** **information provided by** **the clinical team**	15	13.74 (1.61)	3–15	−2.556	**0.002**
**Social support (MSPSS)**					
• Total	6.25	5.89 (1.01)	1–7	−2.339	0.081
• Significant other	6.25	5.86 (1.37)	1–7	−2.194	**0.026**
• Family	6.50	5.97 (1.24)	1–7	−1.408	0.201
• Friends	6.13	5.84 (1.24)	1–7	−0.605	0.586
**Resilience (CD-RISC 10)**	29	29.21 (6.59)	0–40	−0.818	<0.001
**Coping styles (Brief COPE) ^1^**					
• Seeking social support	15.5	16.21 (5.10)	8–32	0.721	**0.006**
• Problem solving	9.5	9.59 (3.31)	4–16	0.594	0.149
• Avoidance	14	14.84 (4.31)	10–40	1.587	**<0.001**
• Positive thinking	16	16.43 (4.30)	6–24	0.205	0.519

B: unstandardised coefficient, *p*-value from simple linear regression. Text in bold indicates *p*-values significant at the *p* < 0.05 level. ^1^ These values are raw scores for each of the coping styles. When data was standardised across the four categories, positive thinking was most commonly used and avoidance least commonly used (see main text).

### 3.3. Multivariable Regression Analysis

The three key independent variables were then combined in a multivariable analysis. Gender and satisfaction with information provided by the clinical team were also included, the former in view of the potential association with anxiety, whilst the latter was significant at the univariable level and was felt to be something clinicians could influence to an appreciable extent. Variables which appeared to have a minimal effect on state anxiety when other factors were accounted for (social support from family, coping through problem solving, and coping through positive thinking) were subsequently excluded from the equation. The final regression model consisted of the variables shown in Table 3, and explained 57.5% of the variation in pre-operative anxiety. Social support from a significant other, resilience, and satisfaction with the information provided by the clinical team retained significance in association with reduced anxiety, whilst avoidance coping remained significantly associated with greater anxiety in the final model.

## 4. Discussion

The baseline characteristics of study participants were generally consistent with those reported in other research involving orthognathic patients [3,22,23,24]. The results suggest that a considerable proportion of patients experience increased pre-operative anxiety, and this can manifest as early as eight weeks before surgery. Interestingly, the median state anxiety score in this study was comparable with that in another orthognathic study where this was assessed two days pre-operatively [3], and this potentially supports having the team intervene from an earlier stage. Gender did not appear to be a major factor affecting pre-operative anxiety. There is interest in the literature relating to the effects of gender on anxiety, in both orthognathic surgery and more general elective surgery [3,25,26,27,28,29]. The current results may however imply less need for tailoring anxiety-reducing interventions in orthognathic surgery patients based on gender. 

As social support has not been explored in detail in relation to orthognathic patients and pre-surgical anxiety, there is little to compare from the orthognathic literature. In the present study, only perceived social support from a significant other was significantly associated with reduced pre-operative anxiety. This was an interesting finding because the significant other subscale refers to a ‘special person’ who the participant turns to for support. It may be that not all members of the patient’s support network are equally effective in providing helpful support, which may explain why the other two subscales (family, friends) did not have a statistically significant relationship with anxiety. It would potentially be beneficial to suggest to patients that they attend pre-operative planning appointments with the person they are most likely to turn to for support during their forthcoming surgery, in order to ensure the clinical team has sufficient opportunity to prepare this individual regarding the best ways in which they can support the patient. Where patients may not have a ‘special person’ providing adequate social support, ways in which to provide additional support could be considered by the patient and the clinical team.

Greater resilience was another factor that was significantly associated with reduced pre-operative anxiety. The median resilience score in this study was slightly lower than that in a general US population (median score: 32), which may suggest potential benefits to improving resilience [30]. It is, however, acknowledged that values derived from a US population may not be entirely reflective of the UK, and comparison with studies in the UK that have also utilised the CD-RISC 10 scale suggest comparable resilience levels with university students [31], a cohort of young adults [32] and a group of older individuals (>55 years old) [33]. There are various resilience-enhancing interventions that have been discussed in the literature [34,35], but it is unclear which of these, if any, may be effective in orthognathic patients. This is an area which should be investigated in more detail in future studies as the pre-surgery orthodontic period may be a useful time to implement such strategies. 

Increased utilisation of avoidance coping was significantly associated with increased state anxiety. This is a form of coping in which an individual changes their behaviour in order to avoid thinking about, feeling, or directly addressing the stressor. Potential signs of avoidance coping that the clinician may be able to recognise include, for example, the patient avoiding asking questions related to the surgery, or stating that they are trying hard not to think about it. Interestingly, previous research suggests that avoidance coping may be associated with reduced pre-operative anxiety [14,15]. To date, however, there does not seem to be much information regarding how coping affects anxiety in orthognathic patients. Contrary to what was hypothesised, increased usage of all four higher-order coping styles was associated with increased pre-operative anxiety. It is difficult to know how the relationship between coping and anxiety actually operates in the pre-surgical period; it may be that patients resort to these coping styles when they feel more anxious, rather than the form of coping contributing to increased anxiety. As patients completed the questionnaires up to eight weeks before the scheduled surgery date, it may also be that their anxiety was just starting to manifest and they had only recently started utilising such coping strategies so there was insufficient time for these measures to have impacted on their degree of anxiety. This potentially explains the discrepancy in results when compared with the literature where anxiety was measured the day before, or even on the day of surgery [14,15]. For coping through seeking social support, it was unclear if patients turned to individuals in their immediate support network or if they utilised connections made through other routes (e.g., on social media) and whether this would have made a difference in relation to anxiety. Future qualitative research may provide deeper insight into the relationship between different coping styles and anxiety in orthognathic patients.

Information provision by the clinical team is a key component of the orthognathic treatment process, and this was also one of the factors considered in relation to anxiety. Previous research in other forms of elective surgery has highlighted the potential for good information provision to reduce anxiety [28,36]. The results of the current study suggest that satisfaction with the information provided was associated with reduced pre-operative anxiety, and further research may provide more detail on specific aspects of information (e.g., content, presentation format) that patients find helpful, such that clinicians delivering the pre-operative information may have a better idea of what information to provide and in what formats. The present orthognathic literature on this aspect is limited, but one other study noted a similar effect of information on anxiety [2]. 

### Study Limitations

Orthognathic cohorts in a given year are generally relatively smaller in number than many other forms of elective surgery, and other studies in the literature have reported similar sample sizes [2,3,37]. Recruitment for this study was also affected by the COVID-19 pandemic when there was a reduction in elective surgery for a considerable time period, and given the circumstances, the present sample is considered to be reflective of the cohort of patients treated. Each of the three units belong to a different organisational unit within the National Health Service, which may potentially make the results more generalisable. It is, however, acknowledged that the final sample size is relatively small in number, which may have implications for the power of the study. 

It should also be noted that the results may be more reflective of the perceptions of Class III patients, given the greater number of Class III patients in the study. However, in previous work, the type of malocclusion was not found to significantly impact on pre-operative anxiety even with a more even distribution of Class II and III patients [3].

## 5. Conclusions

A considerable proportion of orthognathic patients in this study experienced moderate to high levels of anxiety prior to surgery. Based on the final multivariable model, the availability of greater social support from a significant other, greater resilience, and satisfaction with the information provided by the clinical team were factors significantly associated with reduced pre-operative state anxiety, and these may be potential avenues for clinicians to consider in future interventions. Avoidance coping was significantly associated with increased state anxiety, but further qualitative research may provide a better understanding of this relationship.

## Figures and Tables

**Table 1:** Participant characteristics and state anxiety according to participant characteristics (*n* = 70).

		Number of Participants (%)	Pre-Operative State Anxiety (STAI-S) Scores		*p* Value ^1^
			Median	Mean ± SD (Min-Max)	
*Gender*	Male	30 (42.9%)	29.5	34.20 ± 11.37	0.068
				(20–60)	
	Female	40 (57.1%)	35	38.58 ± 10.96	
				(22–63)	
*Ethnicity*	**White**	**33 (47.1%)**	41	39.36 ± 12.52	0.107
				(20–63)	
	**Other ethnic groups**	**37 (52.9%)**	32	34.32 ± 9.58	
	Asian or Asian British	15 (21.4%)		(21–59)	
	Black/African/Caribbean or	8 (11.4%)			
	Black British				
	Mixed ethnic groups	4 (5.7%)			
	Other ethnic group	10 (14.3%)			
*Marital* *status*	Single	65 (92.9%)	35	37.29 ± 11.46	NA ^2^
				(20–63)	
	Married, or in a domestic partnership	4 (5.7%)	30	30.00 ± 2.94	
				(27–33)	
	Separated, divorced, or widowed	1 (1.4%)	NA	-	
*Education* *level*	**Non-university level **	**34 (48.6%)**	35	37.26 ± 10.34	0.462
	GCSE	5 (7.1%)		(23–63)	
	A levels	20 (28.6%)			
	Further education	9 (12.9%)			
	**Degree level**	**36 (51.4%)**	33.5	36.17 ± 12.21	
	Higher education	32 (45.7%)		(20–63)	
	Postgraduate education	4 (5.7%)			
*Employment status*	Student	21 (30%)	32	33.24 ± 9.88	0.104 ^3^
				(20–53)	
	In employment	42 (60%)	35.5	38.05 ± 11.56	
				(21–63)	
	Not currently employed	7 (10%)	39	39.00 ± 12.69	
				(24–60)	
*Type of* *malocclusion*	Class I	3 (4.3%)	43	41.67 ± 12.06	0.711 ^4^
				(29–53)	
	Class II	14 (20%)	35.5	36.79 ± 10.13	
				(26–63)	
	Class III	53 (75.7%)	34	36.40 ± 11.65	
				(20–63)	
*Type of jaw surgery*	**Single jaw**	**25 (35.7%)**	36	37.44 ± 11.87	0.686
	Maxilla only	16 (22.9%)		(20–63)	
	Mandible only	9 (12.9%)			
	**Bimaxillary**	**45 (64.3%)**	33	36.29 ± 11.04	
				(21–63)	

Note: text in bold indicates the major groupings used in the analyses. ^1^ Mann–Whitney U test. ^2^ Too few participants in some categories for meaningful analysis. ^3^ *p*-value compares the anxiety scores between students and those in employment. ^4^ *p*-value compares the anxiety scores between Class II and III patients.

**Table 3:** Parameter estimates from multivariable regression analysis of pre-operative anxiety.

Parameter	B	Std. Error	Beta	t	*p*-Value
Gender *[0 = Male, 1 = Female]*	2.27	2.11	0.10	1.08	0.286
Social support—significant other	−1.57	0.78	−0.19	−2.00	**0.049**
Social support—friends	1.40	0.91	0.15	1.54	0.128
Resilience	−0.40	0.19	−0.23	−2.13	**0.037**
Coping—seeking social support	0.20	0.23	0.09	0.87	0.390
Coping—avoidance	1.19	0.28	0.46	4.31	**<0.001**
Satisfaction with information provided by the clinical team	−1.87	0.59	−0.27	−3.15	**0.003**

B: unstandardised coefficient, Beta: standardised coefficient, *p*-value from multiple linear regression. Text in bold indicates *p*-values significant at the *p* < 0.05 level.

## Data Availability

The data are not publicly available due to ethical and privacy restrictions. Any enquiries regarding the data should be addressed to S. J. Cunningham.
